# Structural and Interfacial Properties of Hyperbranched-Linear Polymer Surfactant

**DOI:** 10.1007/s11743-014-1592-3

**Published:** 2014-05-15

**Authors:** Taotao Qiang, Qiaoqiao Bu, Zhaofeng Huang, Xuechuan Wang

**Affiliations:** 1 Key Laboratory of Auxiliary Chemistry and Technology for Light Chemical Industry, Ministry of Education, Shaanxi University of Science and Technology, Xi’an, 710021 Shaanxi China; 2 Shaanxi Research Institute of Agricultural Products Processing Technology, Xi’an, 710021 Shaanxi China

**Keywords:** Hyperbranched-linear polymer surfactants (HLPS), Hydroxyl-terminated hyperbranched polymer (HBP), Amphiphilic, Structure, Interfacial property

## Abstract

With oleic acid grafting modification, a series of hyperbranched-linear polymer surfactants (HLPS) were prepared by hydroxyl-terminated hyperbranched polymer (HBP), which was gained through a step synthesis method using trimethylolpropane and AB_2_ monomer. The AB_2_ monomers were obtained through the Michael addition reaction of methyl acrylate and diethanol amine. The structures of HLPS were characterised by Fourier transform infrared spectrophotometer and nuclear magnetic resonance (NMR), which indicated that HBP was successfully modified by oleic acid. Furthermore, the properties of surface tension and critical micelle concentration of HLPS solution showed that HLPS can significantly reduce the surface tension of water. The morphology of the HLPS solution was characterised by dynamic light scattering, which revealed that HLPS exhibited a nonmonotonic appearance in particle size at different scattering angles owing to the different replaced linear portions. The relationships of the surface pressure to monolayer area and time were measured using the Langmuir–Blodgett instrument, which showed that the surface tension of monolayer molecules increased with the increasing of hydrophobic groups. In addition, the interface conditions of different replaced HLPS solutions were simulated.

**Electronic supplementary material:**

The online version of this article (doi:10.1007/s11743-014-1592-3) contains supplementary material, which is available to authorized users.

## Introduction

Molecules with dendritic structures contain multiple terminal groups, and they can exhibit numerous properties that linear polymers cannot possess, so these molecules may have great prospects in material, medicine, and some other areas. Compared with linear molecules, molecules with dendritic structures can present some special properties such as lower viscosity, higher solubility, higher reactivity, multiple functional groups, etc. The main structures of dendritic polymers, dendrimers and hyperbranched polymers (HBP) have been showed in previous studies. In order to obtain higher molecular masses, these polymers can be synthesised batch by batch. However, dendrimers, which exhibit a neat structure, are usually expensive or difficult to synthesize, especially when the molar mass is up to the millions. In contrast, HBP, which exhibit a random branch-on-branch topology [1], can be prepared in simple conditions and fewer steps compared with those of the dendrimer [2, 3]. Furthermore, to some extent, HBP also have some properties similar to the dendrimer, such as multiple terminal groups, three-dimensional spatial structures, versatility, and low viscosity. These features of HBP indicate that they can be potentially applied in various industries [4–6].

Surfactants play a crucial role in various accessories, and studies on surfactants have been conducted over the past years. Macromolecular surfactants cannot exhibit satisfactory effects in terms of surface tension, flowability, and viscosity [7]. These defects of macromolecular surfactants may be attributed to the structure of molecules, which can be entangled in solutions [8, 9].

Over the past few years, several researchers have described hyperbranched-linear polymer surfactants (HLPS) and their corresponding interfacial properties in water. Some studies focused on the stability of nanometer gold particles and other metal catalysts in emulsion have been reported [10, 11]. In addition, the encapsulation of drugs and dyes for amphiphilic HBP also have been studied. There were also some papers focused on the properties of the encapsulation and release process of medicine, dye, and metal particles. However, researches concerning the morphology and surface properties of HLPS have not been reported.

According to the synthetic theory of HBP, different generations of hydroxyl-terminated HBP were prepared by changing the mole ratio of AB_2_ type monomer to trimethylolpropane (TMP). The research involved in this paper was conducted using first-generation hydroxyl-terminated HBP modified with oleic acid to obtain six different kinds of structures of HLPS, and the relationship between the structure and properties of the HLPS was determined. Mole ratios of 6:1, 6:2, 6:3, 6:4, 6:5, and 6:6 of the HBP to oleic acid are defined as HLPS-1, HLPS-2, HLPS-3, HLPS-4, HLPS-5, and HLPS-6, respectively. Currently, the surfactants of fatty alcohol polyoxyethylene ether are widely used. However, its synthesis requires harsh conditions, high cost, and elaborate equipment. Thus, in this study, the fatty alcohol polyoxyethylene ether surfactant was replaced by a synthetic HBP nonionic surfactant to produce a novel nonionic surfactant. We focused on the synthesis of a series of HLPS by changing the number of alkyl chains grafted modification on the first generation of HBP. Properties such as interfacial tension of air–water, particle size, and distribution of HLPS in solution were also investigated.

## Experimental

### Materials

Methyl acrylate (MA), which was distilled in advance, was purchased from Tianjin Kermel Chemical Reagent Development Centre (Tianjin, China). Diethanol amine (DEA), *p*-toluenesulfonic acid (*p*-TSA), and TMP were provided by Tianjin No. 1 Chemical Reagent Factory (Tianjin, China). Oleic acid was supplied by Tianjin Kermel Chemical Reagent Development Centre.

### Methods

#### Synthesis of HLP

According to previous studies [12–15] on HBP synthesis, all the processes should be conducted in a dry nitrogen atmosphere. AB_2_ monomer was prepared through the reaction of DEA and MA at 35 °C with constant stirring for 4 h, and methanol was used as the solvent to catalyse the reaction. Then, choosing TMP as the core of HBP, we conducted its reaction with AB_2_ monomer at 120 °C for 3 h to prepare HBP. Finally, the product was purified in a vacuum drying oven at 0.08 MPa and 120 °C for 1 h. Furthermore, HLPS were prepared using a serial ratio of oleic acid to HBP at 130 °C and 0.08 MPa for 3 h.

As sodium oleate generally precipitates in water, the oleic acid cannot react with the hydroxyl group because it will be removed from the system when washing with excess alkali and vacuum filtering. Therefore, the system was distilled until water was completely removed. Then the system was washed with methanol, which can dissolve the HLPS. However, alkali cannot be dissolved in methanol, but could be removed by filtering. Finally, it was distilled to detach methanol from the system and pure HLPS was obtained as the final product.

#### Characterisation by FT-IR

The structures of HLPS were characterised by Fourier transform infrared spectrophotometer (FT-IR) with a Fourier infrared spectrometer (Verte 70, Bruker Daltonic Inc.). ^1^H and ^13^C nuclear magnetic resonance (NMR) were conducted with an AVANVE III (400 MHz) and using *d*-DMSO as the solvent according to the method found in previous studies [16].

#### Surface Tension

Air–water interfacial tension was measured through an automatic surface tension meter (QBZY-1, Shanghai Fangrui instrument Co., Ltd.) by the Wilhelmy plate technique with a sandblasted platinum plate as the sensor. The tension exerted on the sensor was measured by a Beckman microbalance (BS224S, Beijing Sartorius Instrument Co., Ltd.). In addition, every measurement in the whole process was kept in a draft-free plastic cage at 25 ± 0.5 °C; the sensor should be kept in contact with the solutions for 30 min to reach equilibrium [17].

#### Surface Pressure–Area Isotherm

The surface pressure (*π*) of the monolayer was measured with a homemade automatic Wilhelmy balance on a Langmuir–Blodgett instrument (L-B 2000, Nima Inc.) [18]. The surface pressure balance (NIMA 611) has a resolution of 0.1 mN m^−1^. The pressure-measuring system was equipped with a filter paper (Whatman 541, periphery 4 cm). The trough was made from Teflon-coated brass (area 750 cm^2^), and Teflon-made barriers (both hydrophobic and lipophobic) were used in this study. The entire measurement was performed in a draft-free plastic cage at 25 ± 0.5 °C. Moreover, an entire Teflon-made system was constructed to prevent the absorption of materials in the barriers and ensure accurate quantitative analyses until the collapse states of the monolayer of HLPS. During the measurements, the spreading solvent was allowed to evaporate for 15 min before compression and then the monolayer was compressed at a speed of <0.5 nm^2^ min^−1^. At the same time, the instrument recorded the *π*–time isotherm of the sample. The experiment was repeated three times to ensure that the standard deviations for area and surface pressure measurements were below 0.02 nm^2^ and 0.1 mN m^−1^, respectively.

#### Dynamic Light Scattering (DLS)

DLS is used to determine the coefficient of the collective diffusion [19]. The equipment (Brookhaven BI-200SM/BI-9000, Brookhaven Inc.) was composed of a goniometer and a diode laser (coherent Verdi V5, *λ* = 532 nm, maximum output of 5 W), which has single-mode fibre-detection optics. The experiments lasted 5–20 min, and each experiment was repeated three times. The intensity of scattering light was measured at *θ* = 30°, 45°, 60°, 75°, 90°, respectively. The correlation functions from DLS were analysed by the constrained regularised CONTIN method to obtain the distributions of decay rates (Γ); then the distributions of surface mutual diffusion coefficient were calculated [*D* = Γ/*q*2, *q* = (4*nπ*/*λ*)(sin*θ*/2), *n* is the refractive index of the solvent]. The measured correlation function of intensity was related to correlation function in the field, *g* ^(1)^(*q*, *t*), according to the Siegert relationship:

$$ g^{(2)} (q,t) = 1 + [g^{(1)} (q,t)]^{2} $$

The relaxation rate distribution of the system was determined through the CONTIN method with modelling of the correlation function according to following equation:

$$ g_{1} (\tau ) = \int\limits_{0}^{\infty } {G(\varGamma )\exp ( - \varGamma \tau ){\text{d}}\int\limits_{0}^{\infty } {G(\varGamma ){\text{d}}\varGamma = 1} } $$

In addition, the final hydrodynamic radius in surface was calculated according to the Stokes–Einstein equation:

$$ D = \frac{{k_{\text{B}} T}}{3\pi \eta d} $$

where *k*_B_ is the Boltzmann constant, *k*_B_ = 1.38 × 10^−23^, and *η* is the viscosity of water at 26 °C.

## Results and Discussion

### Structure

The FT-IR spectrum can reflect the chemical microstructure of HLPS. The strong bands at 3,300 and 1,366 cm^−1^ could be attributed to the remaining hydroxyl groups. The band at 1,621 cm^−1^ is attributed to the double bond of oleic acid, which indicates that oleic acid has reacted with the hydroxyl groups. The absorption band of the ester group appeared at 1,735 cm^−1^. In addition, the double peaks at 2,925 and 2,854 cm^−1^ correspond to the stretching vibration of –CH_2_–.

The ^1^H and ^13^C NMR spectra indicated the microstructure of the first generation of HPLS. The ^1^H NMR spectrum showed that the shift at 0.9 ppm could be assigned to the hydrogen that came from the TMP (the position in the structure was 59). The shift at 2.0 ppm is attributed to the hydroxyl groups. The strongest peak could be assigned to the –CH_2_– group, which linked with the hydroxyl groups. The small peak at 5.4 ppm belongs to the hydrogen of the double bond in oleic acid, whereas the peak at 2.5 ppm is attributed to *d-*DMSO. The results of the ^13^C NMR spectrum were the same. The peak at 14.36 ppm indicates the carbon on the 60th position of the structure, and the peak at 22.56 ppm is assigned to the carbons at the 52nd to 58th positions; these carbons belong to oleic acid. The most significant peak appeared at 130.05 ppm, which is attributed to the double bond. The hydrogen and carbon spectra indicate that oleic acid has reacted with the hydroxyl group and formed the ester group. Not all the hydroxyl groups were esterified; some of them remained in the HLPS, which is the main target of our future work.

### Water–Air Interfacial Tension for Different Replaced HLPS at Different Concentrations

In order to compare the water–air interfacial tension of the different replaced HLPS, all samples were investigated from high to low concentrations until the interfacial tension increased sharply and became approximately equal. The results indicate that HLPS can considerably reduce the water–air interfacial tension. The results also confirmed that the critical (minimum) micelle concentration (CMC) of HLPS-3 was 3.8 mg/L and the interfacial tension at the CMC was 29.28 mN m^−1^, which were not significantly different from the other HLPS samples. HLPS-3 contains three hydroxyl groups and an alkyl chain, whereas other HLPS samples contain unequal numbers of hydrophobic and hydrophilic groups, which is the main reason for the ability of HLPS to reduce the interfacial tension. These results revealed that HLPS with multiple terminal hydrophobic and hydrophilic groups can significantly decrease the water–air interfacial tension. So the polymers that exhibit a balance of hydrophobic and hydrophilic properties can obviously decrease the surface tension and reach the CMC at a lower concentration.

### DLS for HLPS at Different Scattering Angles

The correlation functions (*C*(*τ*)) of different HLPS at various scattering angles were investigated. However, HLPS-6 could not be measured because of the large oil drip in the water. The *C*(*τ*) of HLPS exhibits an irregular vibration to a large extent when the scattering angle is 90° and 70°; when the scattering angle is 60°, there were also irregular vibrations on HLPS-3, HLPS-4, and HLPS-5. In theory, the distributions of the particle size ratio should achieve a dynamic balance at different scattering regions, and *C*(*τ*) should remain stable. Two reasons may explain the vibrations: first, the concentrations of the samples were extremely low and they caused uneven particle distribution in the solvent. However, the concentrations of HLPS were higher than that of CMC, which demonstrate that this balance cannot be reached. Second, the concentrations of the large particles were also extremely low in the scattering region, so equilibrium could not be achieved because of their slower speed in Brownian movement. This phenomenon could be attributed to the Doppler effect, as revealed in the detecting system. Clearly, the results at different angles showed that the system contains large particles that are unevenly distributed in the solvent, and these particles may result from the impurities of the HLPS or low content in the serial HLPS. From the results, a lower concentration of the large particles was found in the low-replaced HLPS, such as HLPS-6 and HLPS-1.

According to previous studies, the strength of the scattering light on small particles was higher than that of large ones. On the other hand, the smaller particle was easier to detect at larger scattering angles. Based on the Brookhaven BI-200MS system, similar results were obtained at different scattering angles that were isometric to 90°, such as 135° and 45°. From these results, scattering on small particles close to 0° and 180° and scattering on large particle close to 90° were more beneficial for strengthening the light. As scatter on small particles was more effective for increasing the strength of scattering light, so we chose a scattering angle of 45° to reveal the actual properties of the HLPS for the current system.

### Particle Size for Serial of HLPS at Different Scattering Angles

**Fig. 1:**
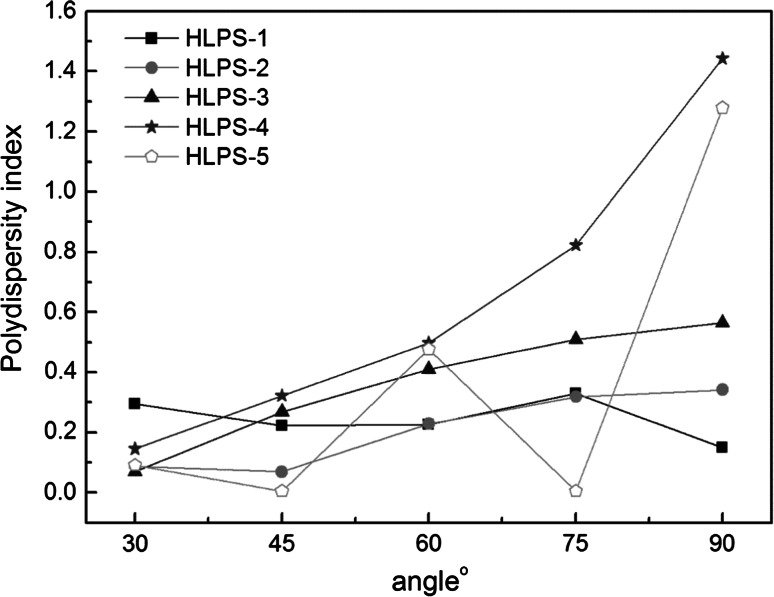
Polydispersity index of a series of HLPS at different scattering angles

As shown in Fig. 1, the polydispersity indexes of HLPS-1, HLPS-2, and HLPS-3 were all lower than 0.7 at scattering angles of 30°, 45°, 60°, 75° and 90°. However, for HLPS-4 and HLPS-5, the polydispersity indices were higher than 0.7 when the scattering angles were 75° and 90°. Previous studies revealed that the system can moderately disperse when the polydispersity coefficient is lower than 0.7, which is the optimum range for DLS. Nevertheless, if it is higher than 0.7, the scattering results may produce errors. However, due to the Brownian movement and uneven distribution of the particle size, different scattering angles can lead to different results for the same sample. The distribution of particle size was investigated. The particle size of HLPS-1 (approximately 200 nm) was smaller than those of other HLPS samples in the solution. This finding could be explained by the fact that only a small portion of the hydroxyl groups can be replaced by oleic acid during the reaction, and it is hard for one HBP to be grafted with several oleic acid molecules at the same time. Therefore, no signal was observed when the particle sizes were larger than 3,000 nm. The results also showed that the particle size of HLPS increased with increasing number of hydrophobic groups. The average particle size of HLPS-2 was approximately 500 nm. Several peaks appeared within the range of size 500–10,000 nm, indicating that the particle size became larger as numbers of multiple-replaced HLPS appeared. This trend would continue with the number of hydrophobic groups increasing. As expected, the average particle size of HLPS-3 increased slowly. It can be speculated that HLPS-3 exhibits a balance of hydrophilic and hydrophobic properties, which can make the particle size reduced. The particle sizes of HLPS-4 and HLPS-5 increased sharply and were distributed over a large range. The instrument did not function properly because of the presence of large oil drips; only when all of the hydroxyl groups were replaced by oleic acid, could it do well. As the number of hydrophobic groups increased, larger particles were unable to dissolve in water, leading to the particle sizes increasing sharply until they could no longer fit into the instrument.

### The Monolayer *π*–Area Isotherm and *π*–Time for Serial HLPS

**Fig. 2:**
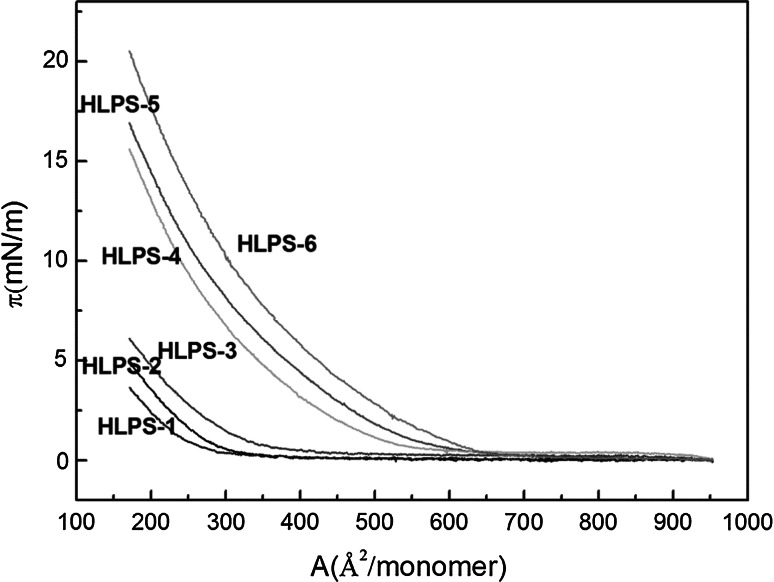
Surface pressure to area isotherm for serial HLPS

The relationship between the monolayer *π* and area isotherm (*π*–*A*) is shown in Fig. 2. As the number of hydroxyl groups replaced by hydrophobic groups increased, the surface pressure and collapse pressure increased. This finding showed that the hydrophilic properties of HLPS decreased with increasing number of hydrophobic groups. The surface pressure of HLPS-6 increased sharply when the monolayer area was 750 Å, while the surface pressure of HLPS-1 increased when the monolayer area was 250 Å. The collapse pressure of HLPS-1 was 4 mN m^−1^, smaller than that of HLPS-6 (27 mN m^−1^). This finding indicated that HLPS-1 was arranged more compactly than HLPS-6. Because HLPS-1 contains more hydroxyl groups, it can adjust to positions to produce a more compact monolayer and decrease the surface pressure. As the number of hydrophobic groups increased, the surface pressure of the monolayer area increased, indicating that the structure was the main reason for the results. The most important reason for this phenomenon may be that the particle size became larger with the increasing number of hydrophobic groups. Also, the surface pressure as well as collapse pressure increased sharply when the monolayer area decreased.

**Fig. 3:**
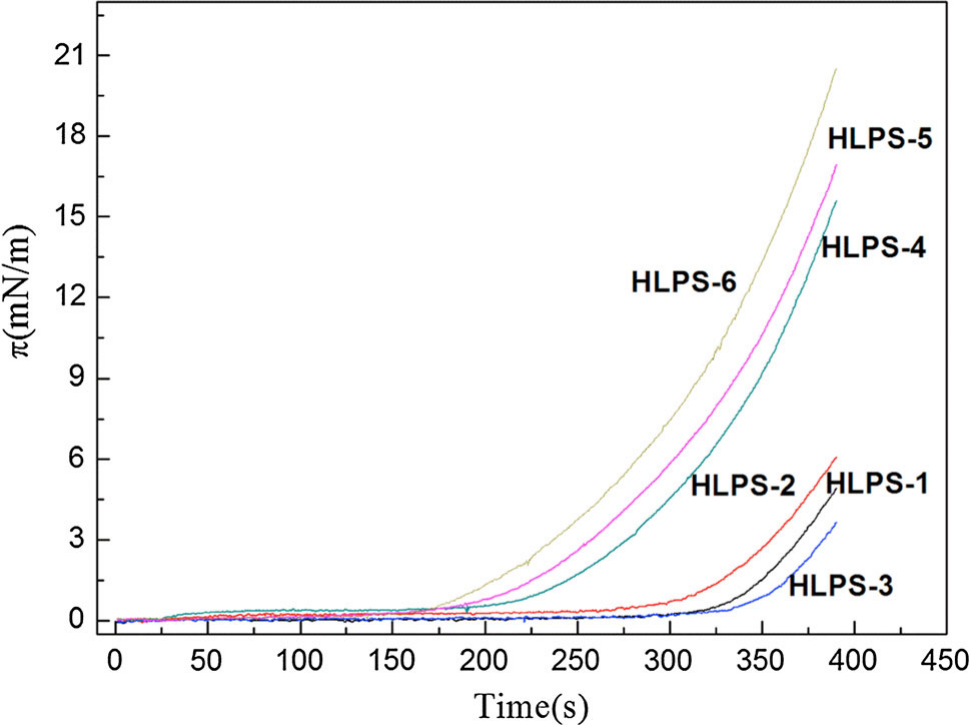
The relationship of surface pressure to time for the series of HLPS

A similar result is shown in Fig. 3. A plot of the surface pressure versus time (*π*–*t*) shows that HLPS-3 needed more time to reach the collapse pressure. At 350 s, the surface pressure began to increase, indicating that HLPS-3 adjusted their molecular positions to the largest extent to avoid collapse. For larger molecules, the surface pressure could be adjusted by the alkyl chains to produce a more compact structure; however, the structure could not become rod-shaped and more compact. As the molecules became larger, more alkyl chains were exposed to air, forming a gap between the molecules and then becoming larger and larger. These changes caused the collapse pressure to increase.

**Fig. 4:**
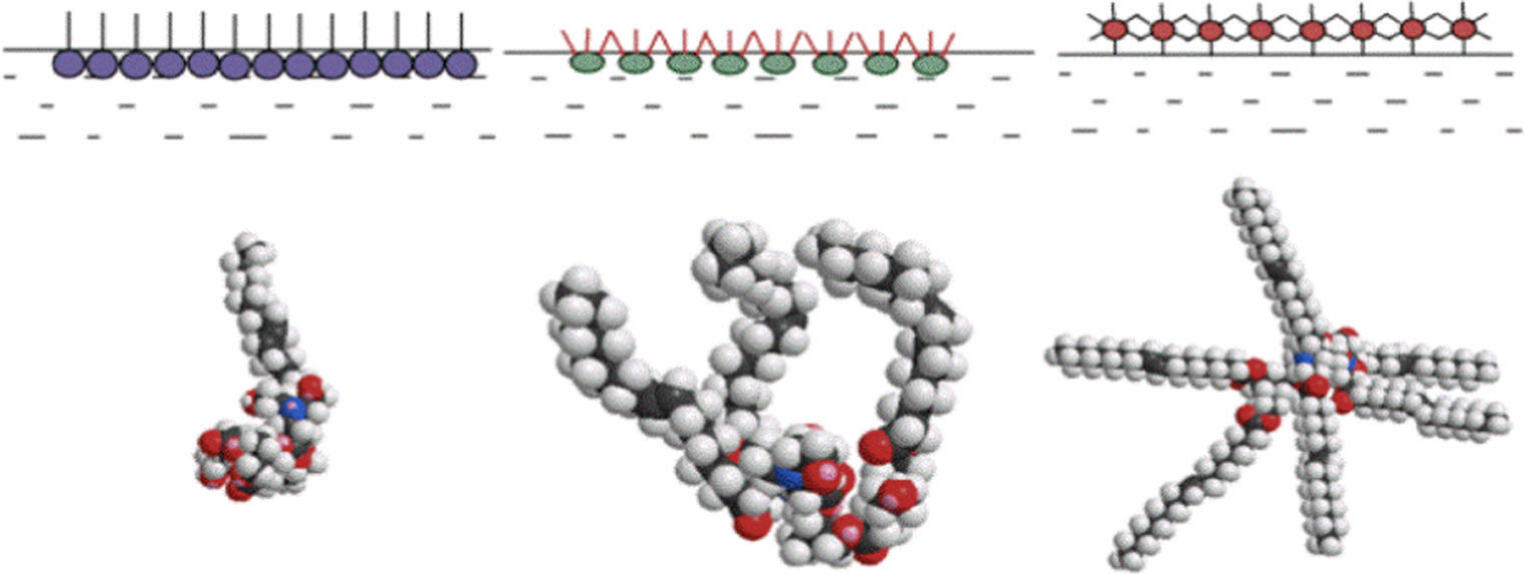
Schematic diagram for HLPS-1, HLPS-3, and HLPS-6 on the interface

We used similar morphologies of HLPS-1, HLPS-3, and HLPS-6 on the interface, based on the results in previous studies (Fig. 4). We inferred that HLPS-1 is arranged as a rod; however, HLPS-6 is arranged as a star, which is unfavourable for obtaining a compact arrangement. HLPS-3 contains three alkyl chains and three hydroxyl groups, which are found arranged at the interface between the rod and star. This arrangement requires more time to adjust to reach the collapse pressure. HLPS-6 did not contain any hydroxyl groups and so could condense as oil drips in water, and the drips were too large to be detected by DLS.

## Conclusions

The AB_2_ monomer was synthesised from the reaction of DEA and MA, and HBP was prepared through the reaction between AB_2_ monomer and TMP. We then esterified HBP with oleic acid to achieve HLPS, and NMR and FT-IR characterisation indicated that the target product HLPS was synthesised successfully.

The results of surface tension measurement showed that HLPS could significantly decrease the surface tension of water and that the CMC of HLPS-3 was the lowest among the CMCs determined. DLS and Langmuir–Blodgett measurements showed that HLPS-3 exhibits special properties in particle size, surface pressure, and collapse time, because of its balance of hydrophobic and hydrophilic properties. With increasing of numbers of replaced hydroxyl groups, the molecules became larger, in turn increasing the performance of larger particle sizes and producing a higher *π* in the monomer layer.

## Electronic supplementary material

Below is the link to the electronic supplementary material.

Supplementary material (DOC 1401 kb)
