# Supplementary material for: Structural and Interfacial Properties of Hyperbranched-Linear Polymer Surfactant
Source: J Surfactants Deterg. 2014 May 15;17(5):959–65. doi: 10.1007/s11743-014-1592-3 (PMC4133636; doi:10.1007/s11743-014-1592-3)
Supplement: Supplementary file 1 — Supplementary material (DOC 1401 kb) [file 11743_2014_1592_MOESM1_ESM.doc]

**Supplementary material**

**Structural and interfacial properties of hyperbranched-linear polymer surfactant**

Taotao Qiang^1,2^, Qiaoqiao Bu^1^, Zhaofeng Huang^1^, Xuechuan Wang^1,2^

1. Key Laboratory of Auxiliary Chemistry & Technology for Light Chemical Industry; Ministry of Education. Shaanxi University of Science and Technology, Xi’an, Shaanxi 710021, China.

2. Shaanxi Research Institute of Agricultural Products Processing Technology, Xi’an 710021 Shaanxi, China

corresponding author email: qiangtaotao@sust.edu.cn

Figure 1. Synthesis of AB_2_ monomer.

Figure 2. Synthesis of hyperbranched polymer

Figure 3. Synthesis of hyperbranched-linear polymers.

.

Figure 4. FT-IR spectrum of HLPS-1.

Figure 5. ^1^H NMR for HLPS-1.

Figure 6. ^13^C NMR for HLPS-1.

Figure 7. Water-air interfacial tension for different replaced HLPS at different concentrations.

(a) HLPS-1 (b) HLPS-2

(c) HLPS-3 (d) HLPS-4

(e) HLPS-5 (f) HLPS-6

Figure 8. Production of a series of replaced hyperbranched-linear polymers.


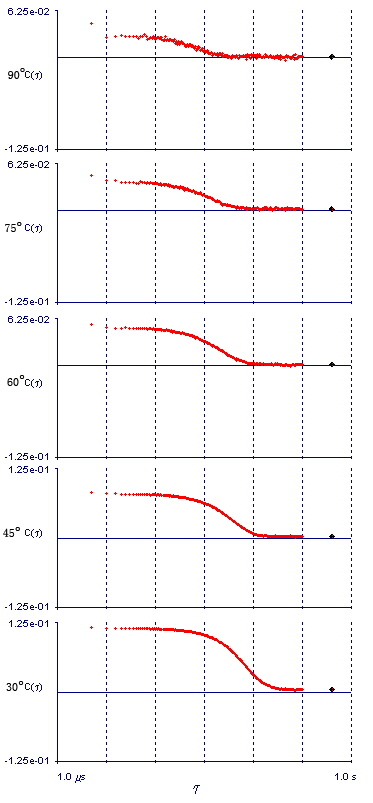

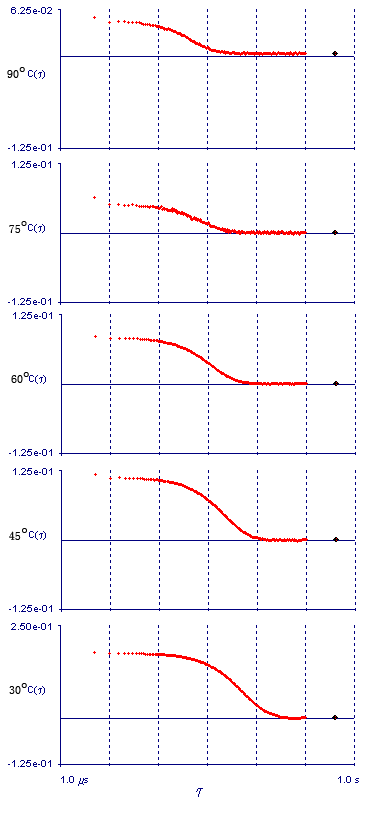

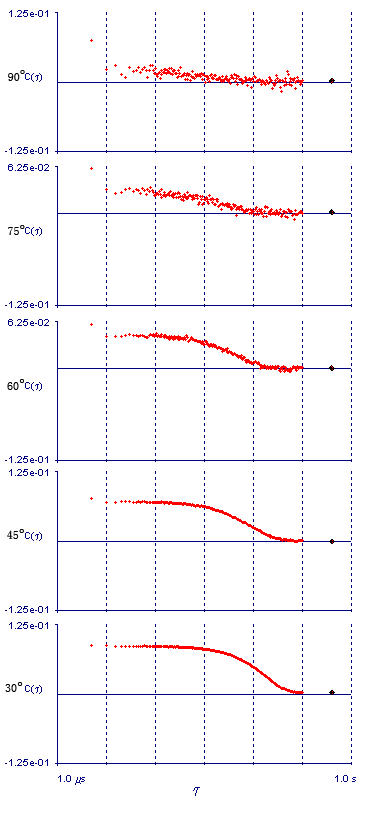


HLPS-1 HLPS-2 HLPS-3


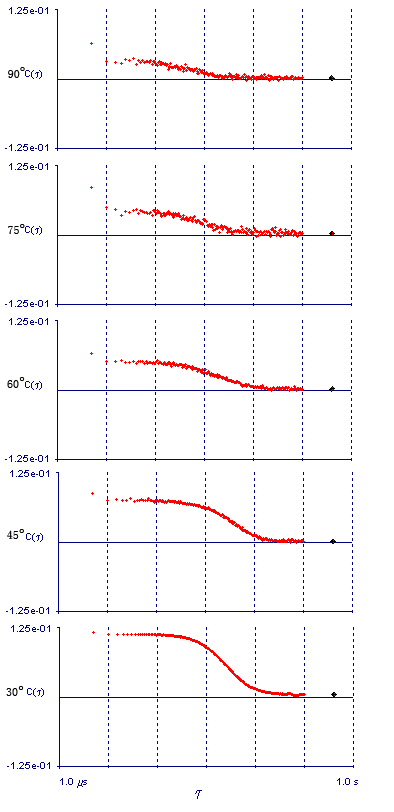

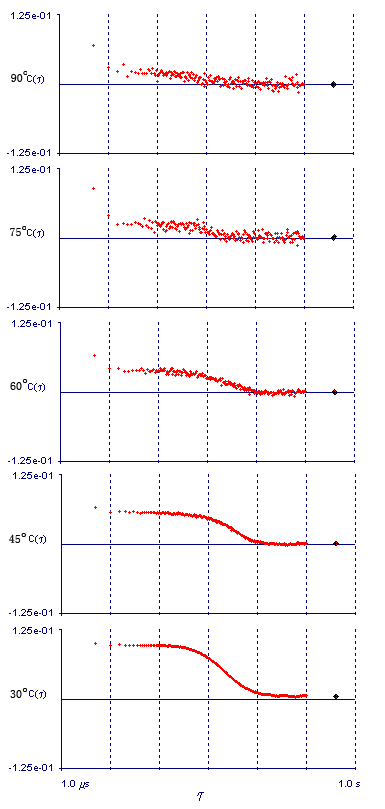


HLPS-4 HLPS-5

Figure 9. Experimental correlation functions for different HLPS at the same concentration.


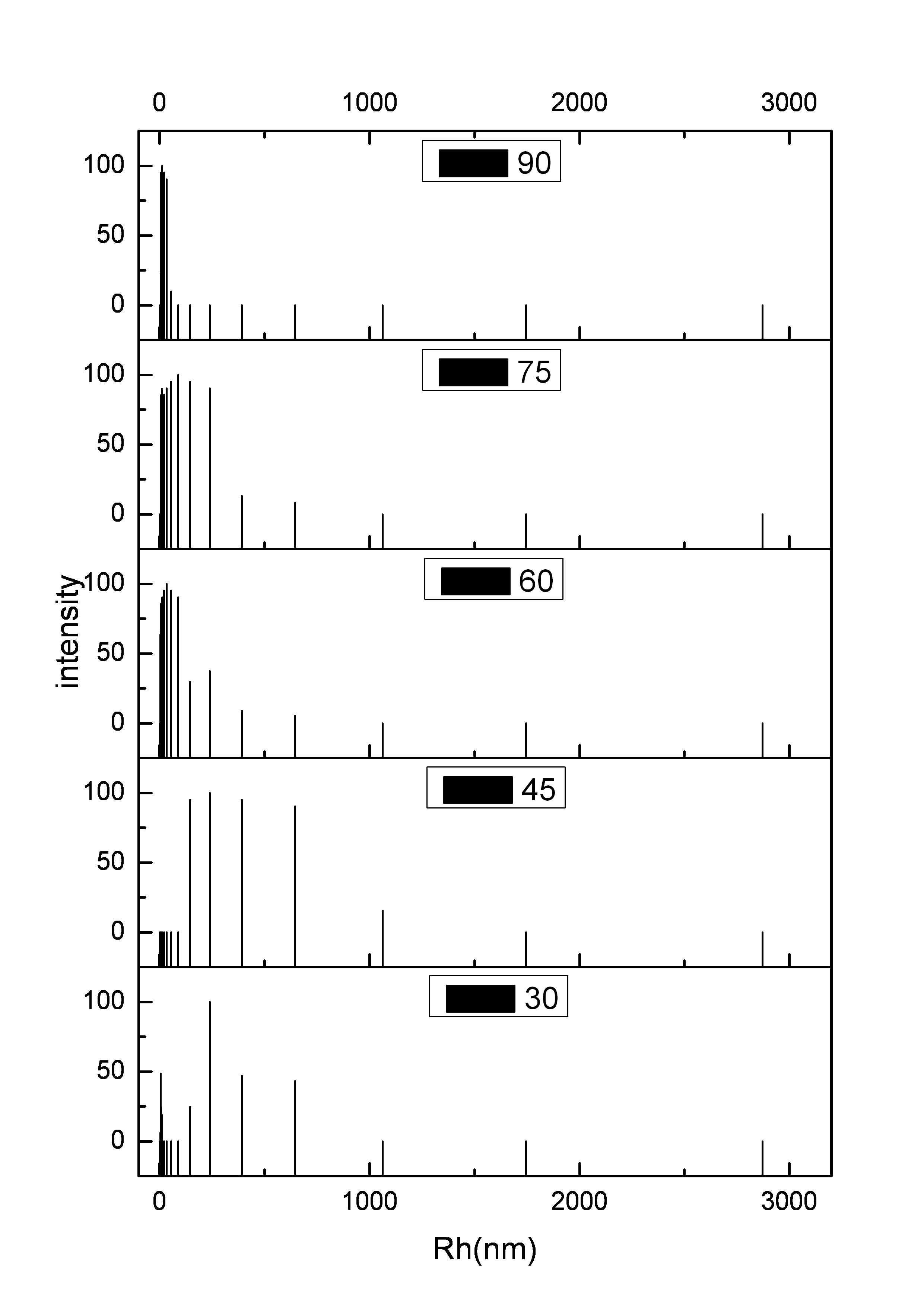

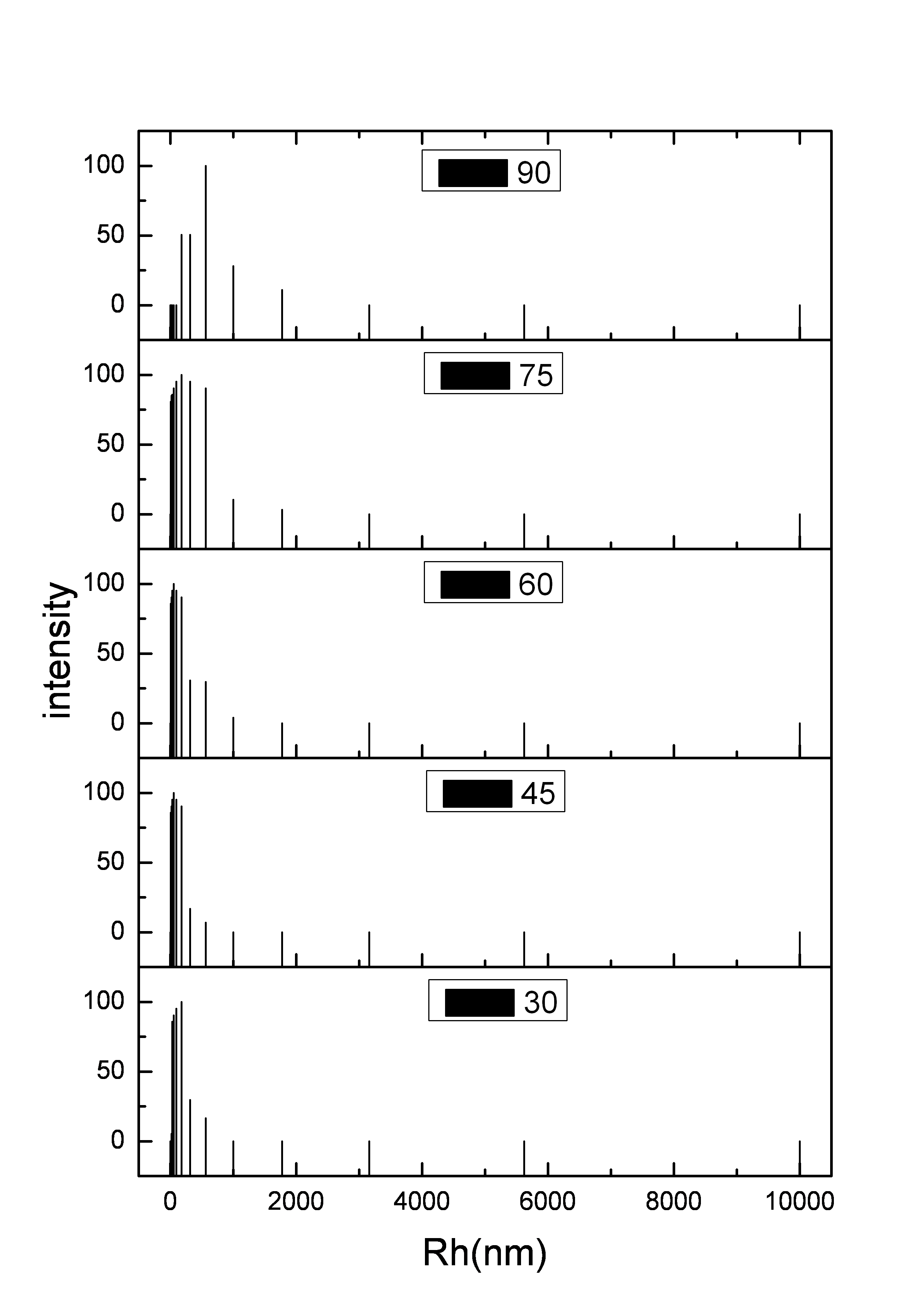

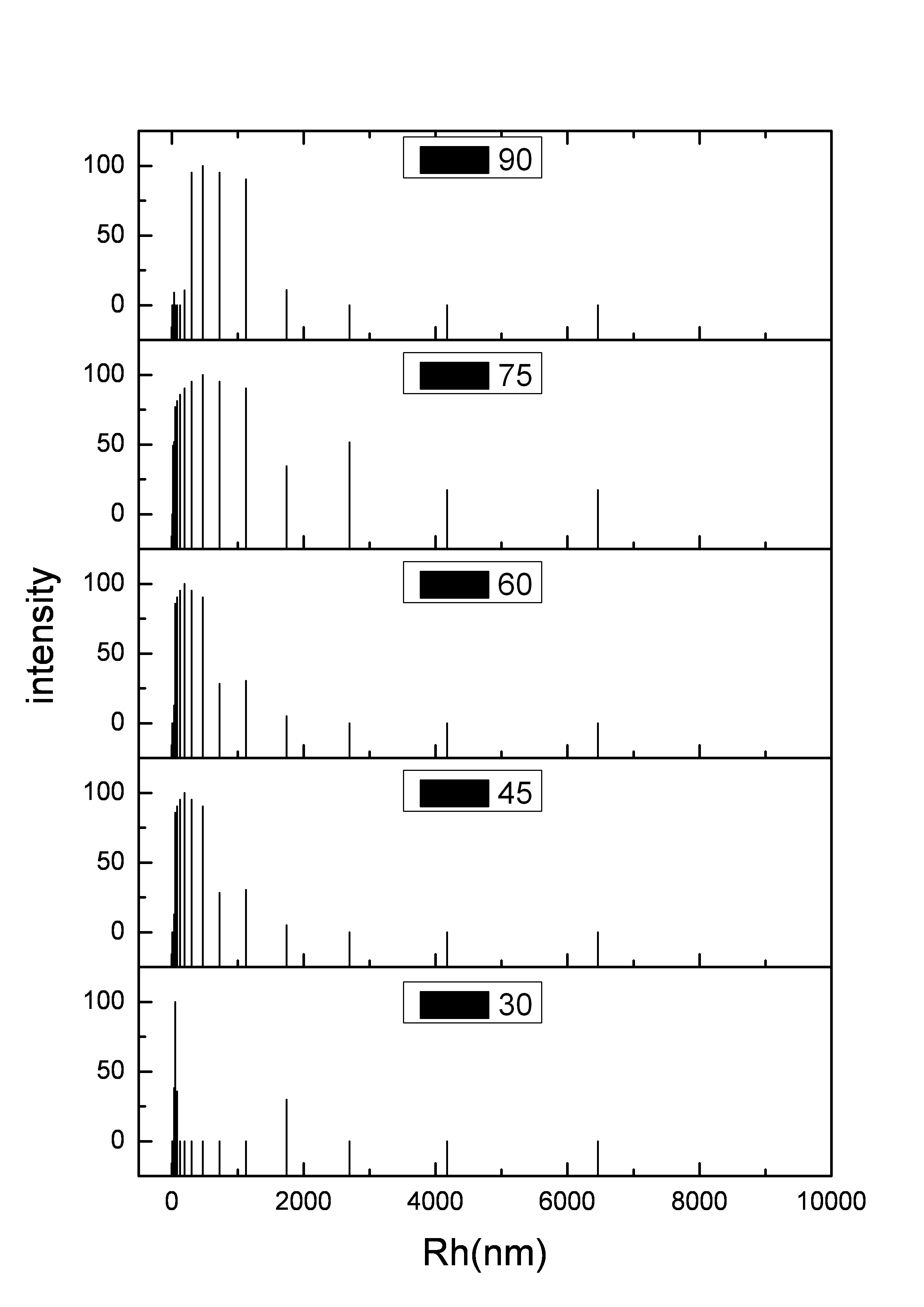


HLPS-1 HLPS-2 HLPS-3


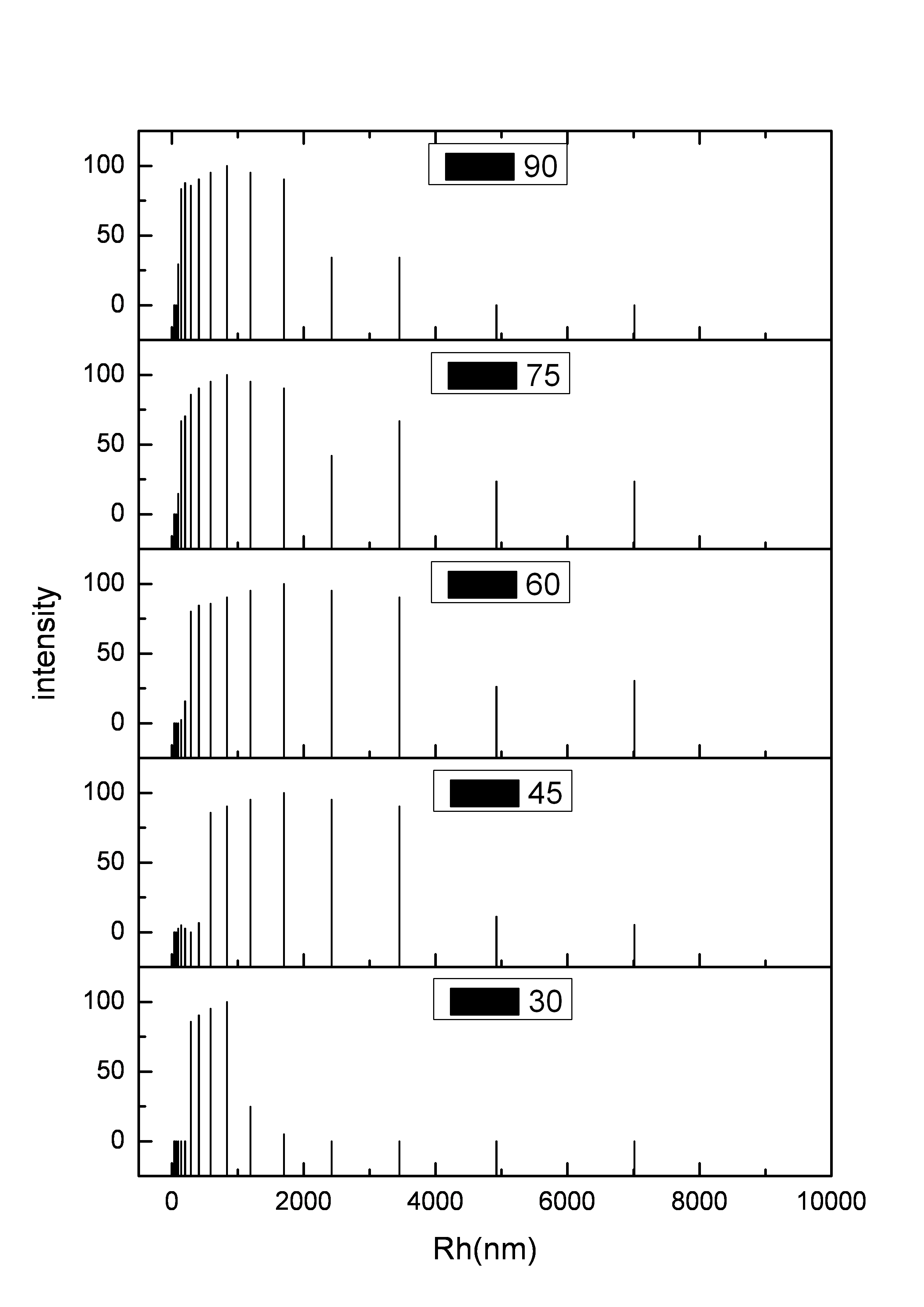

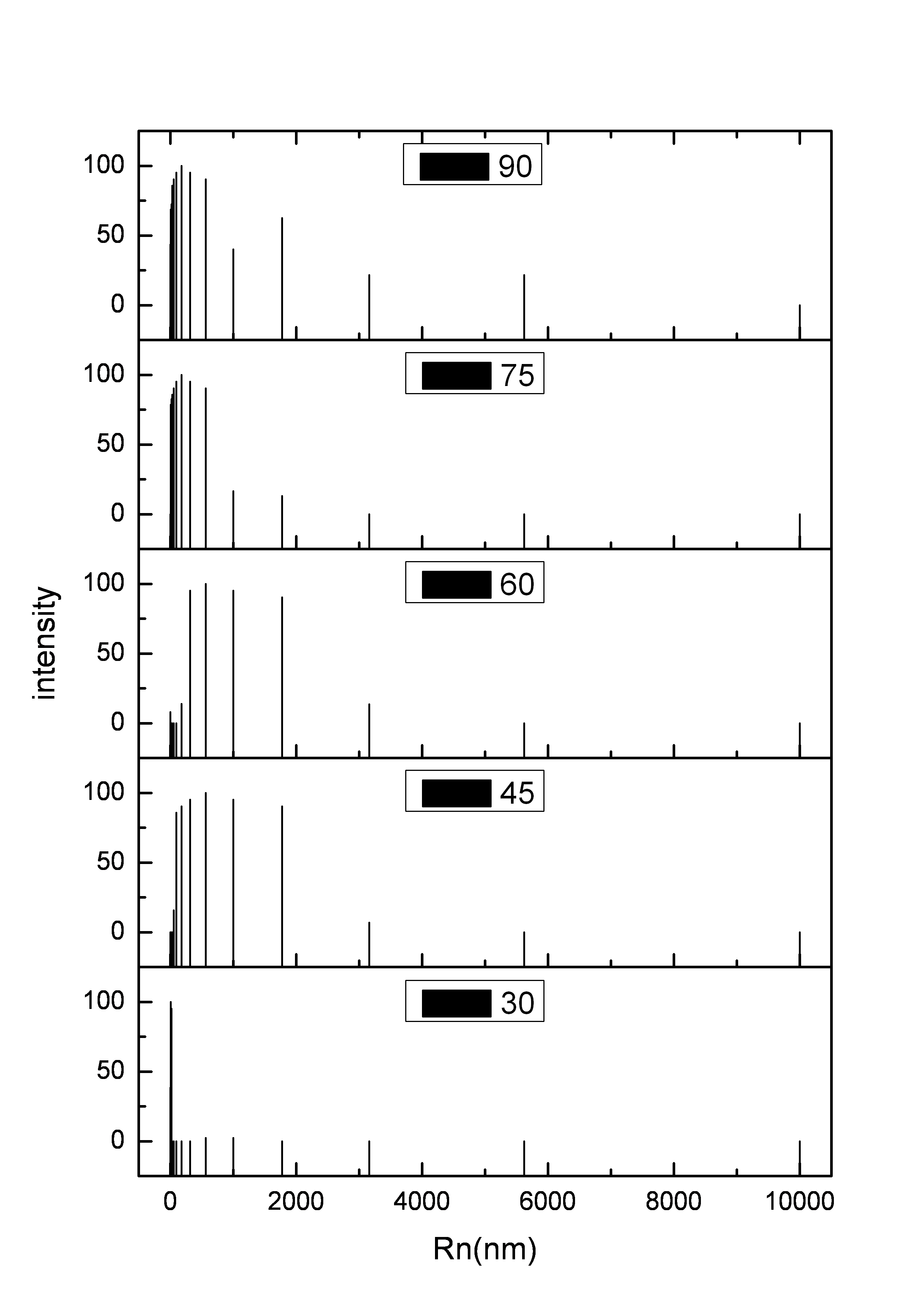


HLPS-4 HLPS-5

Figure 10. Particle sizes of a series of HLPS solutions.
